# Betaine-Based Deep Eutectic Solvents for Phenolic Compounds Extraction from Vine Shoots: Screening, Optimisation, and Cytotoxicity Assessment

**DOI:** 10.3390/foods15203708

**Published:** 2026-10-18

**Authors:** Camila C. Muñoz-Realpe, Carolina Costa, Ana Rita Jesus, María del Mar Contreras, Inmaculada Romero, Eulogio Castro, Ana Rita C. Duarte, Alexandre Paiva

**Affiliations:** 1 Department of Chemical, Environmental and Materials Engineering, Universidad de Jaén, 23071 Jaén, Spain; ccmunoz@ujaen.es (C.C.M.-R.); iromero@ujaen.es (I.R.); ecastro@ujaen.es (E.C.); 2 Institute of Biorefineries Research (I3B), Universidad de Jaén, 23071 Jaén, Spain; 3 LAQV, REQUIMTE, Departamento de Química da Faculdade de Ciências e Tecnologia, Universidade Nova de Lisboa, 2829-516 Caparica, Portugal; acr.costa@campus.fct.unl.pt (C.C.); ar.gameiro@fct.unl.pt (A.R.J.); ard08968@fct.unl.pt (A.R.C.D.)

**Keywords:** agro-industrial waste, antioxidant, betaine, deep eutectic solvents, green solvent, ready-to-use

## Abstract

To develop sustainable extraction of bioactive compounds, this study evaluated the potential of betaine-based deep eutectic solvents (DES). Betaine was applied as a natural, safe hydrogen-bond acceptor in fourteen DES, using polyols/sugars/acids as hydrogen-bond donors to extract vine shoots (VS) phenolic compounds. A conventional solvent, 59% (*v*/*v*) ethanol/water, was used for comparison. Among the tested DES, the extracts obtained with betaine:ethylene glycol:water (1:3:5) and betaine:sorbitol:water (3:1:10) demonstrated high extraction capacity. The betaine:ethylene glycol:water (1:3:5) DES yielded the extract with the most favourable biocompatibility profile and adequate stability at room temperature. The extraction of VS with this DES was optimised using response surface methodology, varying temperature, time, and solid load. It enabled an increase of both the total phenolic content (up to 2.35 mg GAE/mL) and the antioxidant activity (EC_50_ of 162.5 mg extract/mL) under 80 °C, 127 min, and L/S 10:1. Moreover, the DES extract was further profiled by HPLC-UV and HPLC-QTOF-MS, highlighting the occurrence of the high-demand antioxidant *trans*-resveratrol (16 µg/mL) and its dimer *trans*-ε-viniferin (31 µg/mL). To the best of our knowledge, this is the first study to evaluate betaine-based DES for VS extraction, along with its characterisation and cytotoxicity assessment, while discussing their limitations.

## 1. Introduction

Vine shoots (VS) are among the most abundant residues of viticulture, generated during annual pruning, with an estimated production of 2–4 tonnes per hectare [1]. However, despite their wide availability and low cost, their valorisation remains limited. These biowastes consist mainly of a lignocellulosic matrix and contain a relevant fraction of phenolic compounds, particularly stilbenes (e.g., *trans*-resveratrol and *trans-ε*-viniferin) and other metabolites with high antioxidant activity and potential use as functional ingredients [2,3]. Recovering these metabolites is considered a promising strategy to valorise this agricultural residue, enabling the production of high-added-value extracts that support circular economy approaches and the development of natural ingredients for diverse applications [4].

The extraction of bioactive compounds from plant matrices has traditionally been carried out using organic solvents or hydroalcoholic mixtures. Although these systems are effective, they present limitations regarding safety, environmental impact, and solvent recovery for purification. In response, “green extraction” promotes processes that maintain extraction yield and extract quality while minimising the use of hazardous substances and optimising the utilised resources [5]. In recent years, deep eutectic solvents (DES) have emerged as promising alternatives for the extraction of valuable compounds [6]. These eutectic mixtures are typically composed of a hydrogen-bond acceptor (HBA) and a hydrogen-bond donor (HBD) [7]. Their interactions generate a hydrogen-bond network and a marked depression of the melting point relative to the individual constituents, resulting in liquid systems that are stable at room temperature. Their physicochemical properties largely depend on these interactions; therefore, modifying the HBA/HBD ratio or replacing one of the components allows tuning properties such as polarity, viscosity, acidity, and solvating capacity, adapting the system to specific targets [8,9]. DES can be prepared through simple procedures; the most widely used method involves mixing the components under stirring and heating at the selected ratio until a homogeneous liquid phase is obtained [10].

In particular, DES have shown high affinity for phenolic compounds and other bioactive compounds, as they can establish multiple intermolecular interactions both with the target compounds and, in some cases, with components of the plant matrix, thereby promoting their release and solubilisation [10]. From a green chemistry perspective, DES may offer advantages over traditional organic solvents, such as low volatility, low flammability, and high stability. Moreover, depending on their composition, they may exhibit good biodegradability and enable formulations based on constituents with a more favourable safety profile for the intended use [11]. An additional benefit arises when the solvent system is compatible with the final product, allowing the extract to be incorporated together with the DES into the formulation (“ready-to-use” extract). This type of formulation can reduce solvent-removal steps, simplify operational complexity, and lower energy consumption [12], ultimately limiting waste generation. Nevertheless, scaling up DES-based processes poses operational challenges, the most critical being their relatively high viscosity, which can limit mass transfer, compromise extraction efficiency, and increase the energy requirements for mixing and pumping. To mitigate this drawback, strategies such as controlled water addition are used to reduce viscosity and modulate system polarity without compromising the integrity of the eutectic structure [9,13].

Despite the growing interest in using DES for phenolic extraction, solvent selection often prioritises extraction yield, frequently overlooking the systematic incorporation of critical criteria for industrial applicability. Specifically, for extracts intended for sectors in which the solvent remains in the final product, such as food and cosmetic industries, it is essential to evaluate not only the extraction yield but also the chemical and functional stability of the compounds during storage and their biocompatibility profile [14,15]. Systems formulated with betaine (or trimethylglycine) as HBA have been proposed as a relevant option due to their natural origin, typically sourced from sugar beets, and widespread use in consumer products. These formulations represent attractive candidates for designing ready-to-use extracts, combining high solvation capacity with a safety profile aligned with industrial requirements [16], but require case-by-case evaluation.

The objective of this study was to evaluate the potential of several betaine-based DES as a potential alternative to hydroalcoholic solvents for the recovery of antioxidant phenolic compounds from VS. The research systematically integrated solvent screening, safety assessment (biocompatibility in different cell lines), storage-stability evaluation, extraction optimisation, and extract characterisation by high-performance liquid chromatography (HPLC)-UV and HPLC-mass spectrometry (MS). Overall, this work proposes a comprehensive approach for betaine-DES selection in which extraction yield is evaluated alongside safety and stability to preliminarily align with smart, ready-to-use applications. While this betaine-based DES solution widens the portfolio of DES to advance core principles of green chemistry, food systems sustainability, and circular bioeconomy, the limitations have also been discussed.

## 2. Materials and Methods

### 2.1. Raw Material

VS (cultivar ‘Charelo’) were dried at room temperature and ground to ≈1 mm in a ZM 200 ultracentrifugal mill (Retsch, Haan, Germany). The sample was sieved with a 1 mm mesh and stored at room temperature in a dark place. The moisture content was determined gravimetrically, yielding 6.9 ± 0.2%.

### 2.2. DES Preparation

DES were prepared by combining an HBA with an HBD at defined molar ratios. Betaine anhydrous (>97.0%, CAS 107-43-7, TCI, Tokyo, Japan) was used as the HBA, while the HBDs included ethylene glycol (99.5%, CAS 107-21-1, Carlo Erba, Milan, Italy), 1,2-propanediol (or propylene glycol) (≥99.5%, CAS 57-55-6, Merck, Darmstadt, Germany), glucose (≥99.5%, CAS 50-99-7, Sigma-Aldrich, St. Louis, MO, USA), sorbitol (≥98%, CAS 50-70-4, Sigma-Aldrich), lactic acid (~90%, CAS 50-21-5, Sigma-Aldrich), and glycerol (CAS 56-81-5, LabChem, Zelienople, PA, USA). Additionally, ethanol (≥98%, CAS 64-17-5, Honeywell, Charlotte, NC, USA) was mixed with water at a concentration of 59% to be used as a conventional control solvent (control ethanolic extract). Each DES mixture, at the specific HBA:HBD molar ratio, was heated at 50 °C under constant stirring until a homogeneous and transparent liquid was obtained. The DES were then cooled to room temperature and stored in airtight containers, protected from light and moisture, until further use.

### 2.3. Preliminary Study and Extraction Procedures

In the preliminary study, the DES with different compositions and HBA:HBD molar ratios were prepared to evaluate their extraction capacity in terms of the total phenolic content (TPC) and the antioxidant activity against the DPPH radical by measuring the resulting extracts (EC_50_) (Table 1). Extractions were performed in a water bath at 40 °C for 60 min under constant stirring at 400 rpm, using a heating plate equipped with a thermocouple for temperature control. Each extraction was carried out in a tightly sealed flask, with a liquid-to-solid ratio of 20:1, supplemented with 10% (*w*/*w*) distilled water. The extracts were centrifuged at 5000 rpm for 10 min to separate the biomass, and the supernatant was filtered through cotton filters. Samples were stored at 4 °C until analysis. The density of the solvent was determined experimentally at room temperature.

The two DES extracts with the highest content of phenolic compounds and antioxidant activity, together with the control ethanolic extract, were selected to evaluate the stability of the TPC and the antioxidant activity at two conditions (Section 2.5.3) and for cytotoxicity assays in three cell lines (HaCaT, L929, and Caco-2) (Section 2.5.4).

### 2.4. Experimental Design for DES Extraction of Vine Shoots

Based on the preliminary study, the DES that showed the best results in the screening in terms of the TPC, the preliminary antioxidant activity (measured as EC_50_), the storage stability, and the cell viability was selected, i.e., betaine:ethylene glycol:water (1:3:5). A Box–Behnken experimental design was applied to determine the influence of the main factors on the VS extraction using the selected DES-system. This experimental design comprised 17 randomised runs, including four replicates at the central point with five central-point runs. The evaluated factors were temperature (40–80 °C), extraction time (60–180 min), and the liquid-to-solid (L/S) ratio (10:1–30:1; mL DES per g dry biomass). The tested ranges were established according to preliminary studies [17]. The extraction and filtration procedures were carried out as described for the preliminary study. After experimentation, model significance and goodness of fit were assessed by analysis of variance (ANOVA). Process optimisation was performed using response surface methodology (RSM) in Design-Expert^®^ software v12.0.3.0 (Stat-Ease Inc., Minneapolis, MN, USA). Optimal conditions were defined to maximise TPC in terms of concentration, mg gallic acid equivalents (GAE)/mL, and relative to biomass (biomass-referred TPC, mg GAE/g dry VS), and minimise EC_50_ (mg extract/mL). Predicted optimal conditions were experimentally validated in triplicate.

### 2.5. Characterisation of the Extracts

#### 2.5.1. Analysis of Phenolic Compounds

TPC was determined using the Folin–Ciocalteu spectrophotometric method, following the procedure described by Pedras et al. [18]. A gallic acid calibration curve (25–1000 mg/L) was prepared. Samples were analysed in triplicate, and absorbance was measured at 750 nm. Results were expressed as mg GAE/mL (concentration) and mg GAE/g dry VS (relative to biomass).

The identification and quantification of the bioactive phenolic compounds (*trans*-resveratrol and *trans-ε*-viniferin) in the optimal extract was performed by HPLC with UV detection (via diode array detector) (Shimadzu, Kyoto, Japan), following the method described by Muñoz-Realpe et al. [19]. External calibration curves were prepared for *trans*-resveratrol (mg/L): y = 55,984x + 347,526 (R^2^ = 0.99) and *trans*-ε-viniferin (mg/L): y = 28,962x + 315,646 (R^2^ = 0.99). Analyses were conducted using a liquid chromatograph (Shimadzu Corp., Kyoto, Japan) equipped with a C18 BDS Hypersil column (Thermo Scientific, Waltham, MA, USA) of 25 cm in length, 4.6 mm internal diameter, and 5 μm particle size. Moreover, the extract was analysed by HPLC coupled with quadrupole-time-of-flight (QTOF)-MS and MS/MS (Agilent Technologies, Santa Clara, CA, USA) to get insight into the overall phenolic constituents extracted by the selected DES. The electrospray ionisation source was operated in negative ion mode with a continuous infusion of the mass calibrant: trifluoroacetic acid ammonium salt and hexakis 1H,1H,3H–tetrafluoropropoxy) phosphazine (Agilent Technologies). The analysis and characterisation work were carried out according to previous work on VS [2,19] using the Agilent MassHunter Qualitative Analysis software B.06.00; the resulting data were compared with these reference works.

#### 2.5.2. Antioxidant Activity

Antioxidant activity was assessed using the DPPH (2,2-diphenyl-1-picrylhydrazyl) colourimetric assay, following the procedure described by Pedras et al. [18]. Absorbance was measured at 517 nm, and all assays were performed in triplicate. The initial DPPH solution was adjusted to an absorbance range of 1.0–1.1, and distilled water was used as the blank. The percentage of inhibition was calculated using Equation (1), where A_DPPH_ corresponds to the absorbance of the blank and A_sample_ to the absorbance of the samples.

(1) $$\%\;inhibition=\frac{{(A}_{DPPH}-A_{sample})}{A_{DPPH}}$$

Then, EC_50_ values were determined from the inhibition curves obtained using serial dilutions of the extracts. Results were expressed as EC_50_ (mg extract/mL), defined as the DES extract concentration required to scavenge 50% of the radicals present in the assay.

In addition, the colourimetric methods, ferric ion reducing antioxidant power (FRAP) and ABTS (2,2′-azino-di (3-ethylbenzothiazoline-6-sulphonic acid)) assays were carried out at 593 nm and 734 nm, respectively, in a Bio-Rad iMark™ microplate reader (Hercules, CA, USA). The antioxidant Trolox (6-hydroxy-2,5,7,8-tetramethylchroman-2-carboxylic acid) (VWR Chemical; Gliwice, Poland) was used to build the calibration curve for each assay, and the results were expressed as milligrams of Trolox equivalent (TE) per gram of dry VS [19]. The DES mixtures were tested in these assays to confirm that there was no influence on the colourimetric methods.

#### 2.5.3. Stability Tests

The stability tests were carried out under different storage conditions: room temperature with exposure to natural light, and 40 °C in the dark (in an oven). The TPC and antioxidant activity were measured in the extracts at time intervals of 0, 7, 14, 30, and 60 days in order to evaluate the effect of the temperature on their stability. TPC was determined using the Folin–Ciocalteu method (Section 2.5.1), while antioxidant activity was determined using the DPPH assay, as described in Section 2.5.2. All data were expressed as the mean ± standard deviation (SD) (*n* = 3) and, for each assay type, the means were compared with their respective 0-day sample using ANOVA and the Dunnett’s post hoc test (GraphPad Prism, v. 11.0.0; GraphPad Software, San Diego, CA, USA).

#### 2.5.4. Cytotoxicity Studies

The cytotoxic effects of the extracts were evaluated using the CellTiter 96^®^ AQ_ueous_ One Solution Cell Proliferation Assay (Promega) (Madison, WI, USA), an MTS-based method (3-(4,5-dimethylthiazol-2-yl)-5-(3-carboxymethoxyphenyl)-2-(4-sulfophenyl)-2H-tetrazolium). Human keratinocytes (HaCaT; CLS Cell Lines Service GmbH, Eppelheim, Germany), mouse fibroblasts (L929; Leibniz-Institut DSMZ, Braunschweig, Germany), and human colorectal adenocarcinoma cells (Caco-2; Leibniz-Institut DSMZ) were used. Cells were incubated at 37 °C and 5% CO_2_ in 96-well plates at seeding densities of 4.5 × 10^5^ cells/well (3 days), 1 × 10^5^ cells/well (1 day), and 2 × 10^5^ cells/well (8 days), respectively. Subsequently, cells were exposed for 24 h to different concentrations of DES extracts (12.5–200 mg extract/mL) and ethanol/water extract (12.5–200 μL/mL) prepared in culture medium under the same conditions. Cells incubated with culture medium alone were used as the negative control. After exposure, 100 μL of MTS reagent was added to each well and plates were incubated for an additional 3 h. Absorbance was measured at 490 nm using a microplate reader (Infinite 200 PRO MPlex) (Tecan, Männedorf, Switzerland), and cell viability was calculated using Equation (2), where A_sample_ corresponds to sample absorbance and A_control_ to the negative control absorbance. Three independent experiments were conducted, each evaluated in triplicate.

(2) $$\%\;Cell\;viability=\frac{A_{sample}}{A_{control}}\times 100$$

All data are mean ± SD (*n* = 3). As the methodology applied to measure the cytotoxicity assays with DES has to take into account the density because they are viscous, the concentration was expressed in mg/mL (mg extract/mL). For ethanol/water extracts, it was expressed in μL/mL or approximated to mg/mL using the theoretical density of the aqueous ethanolic solution. For each cell line, a two-way ANOVA was applied to evaluate the effects of extract type, concentration, and their interaction on cell viability. Subsequently, for each extract, the different concentrations were compared with the negative control (culture media) using the Dunnett’s post hoc test (adjusted *p*-values) (GraphPad Prism).

## 3. Results and Discussion

### 3.1. Screening of DES Systems for Phenolic Extraction

DES can extract metabolites spanning a wide range of polarities and, owing to their ability to form extensive hydrogen-bonding networks, are particularly suitable for recovering phenolic compounds from plant matrices [10]. The extraction capacity of DES depends on multiple variables, including water content, polarity, viscosity, and the HBD:HBA molar ratio [9]. To assess their potential, fourteen DES systems (Table 1) were evaluated using betaine as HBA and polyols (ethylene glycol, propylene glycol, glycerol, and sorbitol), acids (lactic acid), and sugars (glucose) as HBD. All extractions were realised under identical experimental conditions, evaluating TPC and antioxidant activity against the DPPH (EC_50_) and ABTS radicals as response parameters, along with FRAP (Table 2). 10% (*w*/*w*) water was added to all systems after preparation, as the addition of water can favour the extraction of bioactive compounds, without significantly compromising the molecular interactions between the HBA and HBD [20,21]. Moreover, since reducing particle size can improve extraction capacity by increasing the available surface area while favouring homogeneity, VS were milled and sieved to an approximate particle size of 1 mm [22].

The ethanolic extract control showed a phenolic content of 0.80 mg GAE/mL (16.4 mg GAE/g dry VS) and an EC_50_ of 625.8 mg extract/mL, and was used as a reference for comparing the yield of the evaluated DES systems. This solvent (59% ethanol/water solution) was selected based on a previous study [19], which suggested that intermediate levels of ethanol are adequate for solubilising phenolic compounds from VS, particularly *trans*-resveratrol and *trans*-*ε*-viniferin. The data in Table 2 showed that, depending on the DES formulation, the TPC of VS extracts (Table 2) ranged from 0.41 to 0.82 mg GAE/mL, corresponding to 9.4 and 18.9 mg GAE/g dry VS, respectively. The antioxidant activity, expressed as EC_50_, ranged from 611.2 to 1522.1 mg extract/mL. The FRAP and ABTS results varied between 3.8 and 8.6 TE/g dry VS and 17.0 and 56.7 TE/g dry VS. Based on the extraction efficiency and the antioxidant activity data, the results confirm that some of the tested DES constitute promising alternatives to ethanol/water for extracting phenolic compounds from VS. In particular, the betaine:ethylene glycol:water systems at 1:3:5 and 1:3:10 (DES 3 and 4 extracts, respectively) achieved the highest TPC values (≈0.8 mg GAE/mL; ≈19 mg GAE/g dry VS) and the best antioxidant activity (e.g., EC_50_ about 611 and 626 mg extract/mL, respectively). These values were comparable to the ethanolic control (Table 2).

Previous studies have applied choline chloride (ChCl) as HBA in DES formulation to extract phenolic compounds from VS. For example, Duarte et al. [23] demonstrated a higher extraction capacity of an aqueous solution of levulinic acid/ChCl compared to the 80% methanol control, with a total phenolic yield of about 12 versus 1 mg/g dry weight, respectively. Meanwhile, Mattonai et al. [24] reported a higher combined concentration of resveratrol and catechin in extracts using ChCl-polyol-based DES (≈ 330–350 ppm) compared to hydroethanolic extracts (≈210 ppm) from VS waste. Using ChCl:ethylene glycol at 85 °C, the yield varied from 12 to 22 mg/g, depending on the cultivar. These studies focus on different DES systems, some of which may have practical limitations due to regulatory restrictions or high costs [25,26]. Alternatively, this work provides an alternative based on betaine as HBA, and it shows that adjusting the water content (e.g., DES 3/4 vs. DES 1) allows for matching or improving yield relative to ethanol/water (Table 2) or comparable to that of the aforementioned studies. Particularly, given the applicability of DES in the cosmetic, pharmaceutical, and food industries, this study exclusively employed betaine owing to its natural and renewable origin, safety, and security; e.g., it has a positive opinion by the EFSA [27], and it is considered a compatible component for cosmetics [28].

The presence of water in the betaine:ethylene glycol DES formulation improved the extraction of phenolic compounds from VS. With these DES combinations, an increase of 31% in TPC was observed from DES 1 (1:3) to DES 4 (1:3:10) extracts, rising from 0.62 to 0.81 mg GAE/mL and a decrease in EC_50_ from 709.5 to 625.9 mg extract/mL, which means an increase of the antioxidant activity (e.g., the EC_50_ decreased about 12%). This behaviour may be explained by the effect of water on the physicochemical properties of the DES, particularly by reducing viscosity and modulating polarity, which enhances mass transfer and the diffusion of phenolic compounds from the plant matrix. Moreover, the pH can also be modified, which can also affect the extraction efficiency [29,30]. For example, studies performed by Wang et al. [31] on *Celtis sinensis* leaves reported maximum extraction yields in DES containing 30–50% (*w*/*w*) water, while Petit et al. [32] found that adding 20–40% (*w*/*w*) water improved antioxidant activity without affecting TPC in VS extracts obtained with ChCl- and polyol-based DES, with an optimum close to 38%. Considering the compositional analysis reported by Rodrigues et al. [30], the water content of DES 3 and 4 is within this range (about 24% and 38%, respectively), which could generally be favourable for extracting antioxidants, according to the latter studies. However, the positive effect of the water content in the DES formulation was not observed in some cases (e.g., DES 5 and DES 6).

Moreover, systems based on 1,2-propanediol (DES 5 and 6 extracts), lactic acid (DES 7 and 8 extracts), glycerol (DES 12 and 13 extracts), and glucose (DES 14 extract) showed lower phenolic concentrations (≤0.78 mg GAE/mL) and antioxidant activity in the three tests (e.g., higher EC_50_ values ≥955 mg extract/mL) than extracts obtained with betaine-ethylene glycol DES combinations or the ethanolic/water control extract. These differences may be related to variations in the physicochemical properties of the medium and in the affinity of phenolic compounds for the solvent. DES containing sorbitol (DES 9–11 extracts) showed an intermediate yield, with phenolic contents of approximately 0.51–0.59 mg GAE/mL and moderate antioxidant activity (EC_50_ varied between 835 and 929 mg extract/mL). Overall, these findings suggest that antioxidant activity is determined not solely by TPC but also by the profile of the extracted compounds. For instance, DES 8 extract exhibited a relatively high TPC (0.78 mg GAE/mL) but a high EC_50_ (955.6 mg extract/mL), whereas DES 9 extract has an intermediate TPC value (0.59 mg GAE/mL) and a lower EC_50_ (835.3 mg extract/mL). The study on VS reported by Petit et al. [32] indicates that, even when DES extracts display similar TPC values, antioxidant activity can differ, supporting the influence of the phenolic profile on bioactivity. In that study, the ChCl:1,4-butanediol extract exhibited the highest antioxidant activity, which was related to a higher proportion of stilbenes (*trans*-resveratrol and *trans-ε*-viniferin) in the extract.

In silico studies by Kiene et al. [20] reported enhanced solubilisation of major VS phenolics (viniferin and resveratrol) in DES formulated with ChCl as the HBA and polyols as the HBD (1:1 molar ratio). Other studies have shown that polyalcohol-based DES exhibit a higher extraction capacity for resveratrol from peanut roots than DES based on organic acids, which has been attributed to differences in hydrogen-bonding capacity and favourable thermodynamic parameters for solvation, as well as a polarity better suited to a polyhydroxylated polyphenol such as resveratrol [21,22]. In addition, these studies indicate that incorporating 10–30% water can enhance stilbene extraction by modulating DES structure and improving solute mobility and accessibility [20,21].

Overall, the formulations with betaine-ethylene glycol-water (1:3:5) (DES 3 extract) and betaine-sorbitol-water (3:1:10) (DES 9 extract) were selected for further studies of cytotoxicity and stability, as these combinations had higher TPC and/or lower EC_50_ values. Compared with the DES 9 extract, the DES 3 extract showed an approximate 40% increase in the TPC value and a 37% reduction in the EC_50_ value. The analysis by HPLC-UV at 280 nm revealed that the phenolic profiles were quite similar, but with quantitative differences (Figure S1). Nevertheless, sorbitol offers a green, low-toxicity, and biodegradable component in the betaine DES system for extracting VS phenolic compounds. This compound is generally recognised as safe (GRAS) for human consumption and used in cosmetics, which makes it a suitable ingredient for food, cosmetic, and pharmaceutical applications [6].

### 3.2. Cytotoxicity of the Selected Extracts

DES have gained attention as potentially safer and more sustainable alternatives to conventional organic solvents, increasing their attractiveness for food, cosmetic, and pharmaceutical applications [33,34]. However, their safety depends on the composition, the HBA:HBD ratio, and the water content of each formulation; therefore, cytotoxicity assessment is essential [35]. Based on the preliminary evaluation of the fourteen DES-based extracts (Section 3.1), the DES 3 and DES 9 extracts were selected to assess their cytotoxicity, along with the control ethanolic extract. Evaluation was performed using three different cell lines: HaCaT (immortalised human keratinocytes) as a model for dermal toxicity; L929 (mouse fibroblasts) as an indicator of connective tissue compatibility; and Caco-2 (human intestinal epithelial cells) as a model of the intestinal epithelium [30,36]. Overall, this panel enabled a comprehensive assessment of the cytotoxicity of the selected extracts. Cell viability was expressed as a percentage relative to the negative control. Results were interpreted according to EN ISO 10993-5, considering a potentially cytotoxic effect when viability was below 70% of the control [37].

**Figure 1:**
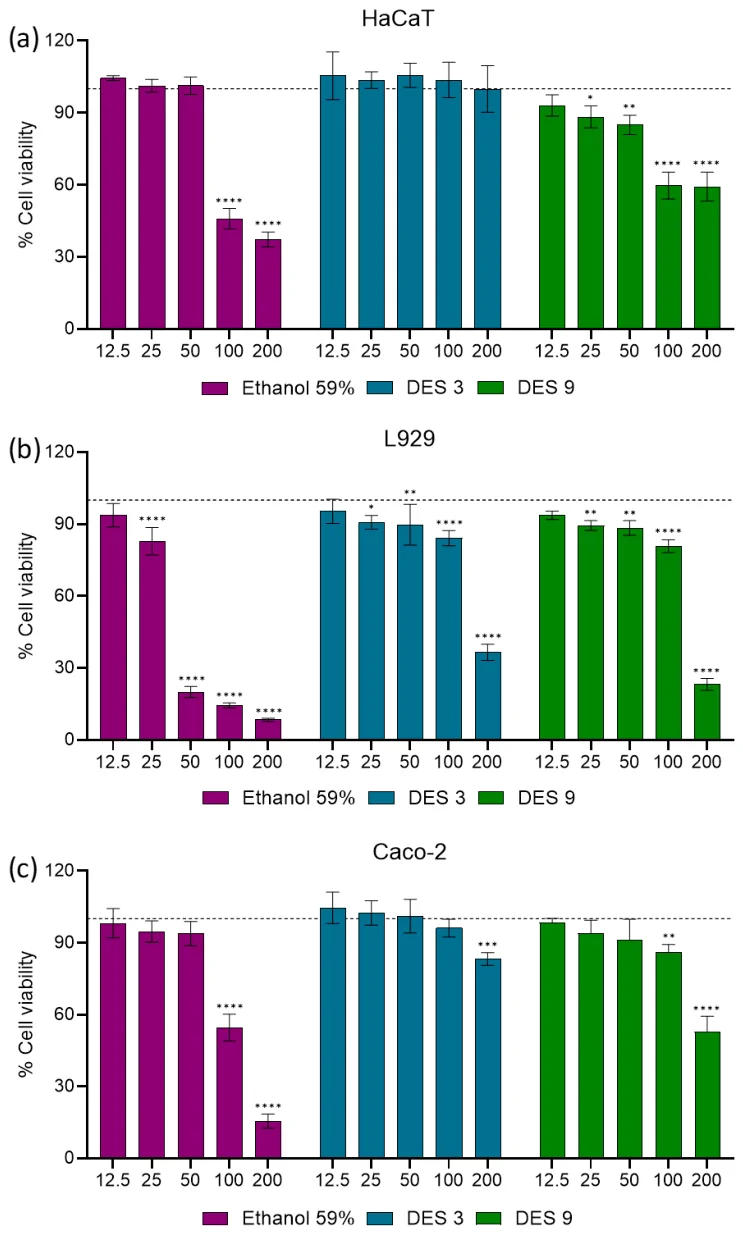
Cell viability after 24 h exposure to ethanol/water (59%, *v*/*v*), DES 3, and DES 9 extracts in (**a**) HaCaT, (**b**) L929, and (**c**) Caco-2 cells. Doses: ethanol/water extract (12.5–200 μL/mL); DES 3 and DES 9 extracts (12.5–200 mg extract/mL). The dashed line represents 100% viability (negative control). The means were compared by two-way ANOVA with Dunnett’s post hoc test with the negative control (* *p* < 0.05, ** *p* < 0.01, *** *p* < 0.001, **** *p* < 0.0001).

In HaCaT cells (Figure 1a), the control ethanolic extract maintained viabilities close to 100% up to 50 μL/mL (*p* > 0.05), but showed a significant reduction at 100 and 200 μL/mL (about 91 and 182 mg extract/mL) (45.9% and 37.3%, respectively), indicating potential cytotoxicity at high concentrations (*p* < 0.05). The DES 3 extract showed no relevant changes relative to the control at any of the tested concentrations, with a viability of 99.9% even at 200 mg extract/mL (*p* > 0.05). In contrast, the DES 9 extract exhibited a progressive decrease, with viabilities below 60% at 100 and 200 mg extract/mL (*p* < 0.05). For L929 cells (Figure 1b), the ethanol/water extract showed the highest cytotoxicity, with a significant decrease (*p* < 0.05) in viability at 50 (20.0%), 100 (14.4%), and 200 μL/mL (8.4%). For the DES 3 and DES 9 extracts, viability remained above 70% up to 100 mg extract/mL (84.2% and 80.8%, respectively), whereas at 200 mg extract/mL both decreased to 36.6% and 23.1%, respectively, indicating potential cytotoxicity only at the highest concentration (*p* < 0.05). In Caco-2 cells (Figure 1c), the ethanolic extract maintained viability above 93.8% up to 50 μL/mL (*p* > 0.05), decreasing to 54.6% and 15.5% at 100 and 200 μL/mL (*p* < 0.05), respectively. The DES 3 extract was again the least cytotoxic, maintaining the viability to about 96% at 100 mg extract/mL (*p* > 0.05) and 83.2% at 200 mg/mL (*p* < 0.05). By contrast, DES 9 extract showed lower viability values at these concentrations.

These results suggest that the cytotoxicity of the extracts depends on both the cell line and the extraction solvent, including the DES composition. Previous studies have evaluated both the solvents and DES-derived extracts, highlighting the importance of DES composition in biocompatibility assessments and cell viability [15,35,38]. In this line, the present results suggest that the ethanolic extract exhibited the most cytotoxic profile, with viabilities below 70% in all three cell lines at high concentrations. Among the DES extracts, DES 3 showed the highest biocompatibility, maintaining viabilities above 70% in HaCaT and Caco-2 across the entire tested range and dropping below 70% only in L929 at 200 mg extract/mL. In contrast, DES 9 showed lower viability at high concentrations in the three cell lines studied, suggesting lower tolerance compared with DES 3. Therefore, switching the HBD from ethylene glycol to sorbitol was associated with differences in cell viability at high concentrations, indicating that the observed cytotoxic response may be influenced by both the DES formulation and the composition of the resulting extracts. This agrees with a previous study on Caco-2 that reported different bioactivity and cytotoxicity depending on the betaine-DES formulation, including the HBD type and the water content [30]. For example, these authors found that for studies with betaine/polyol-based DES reported EC_50_ values of 103.5–259.6 mg/mL, including an EC_50_ of 167.1 mg/mL for a betaine-ethylene glycol-water system (1:3:5.2), and also observed a trend toward lower cytotoxicity with increasing water content [30]. Other authors reported Caco-2 EC_50_ values for hydrophobic DES in the range of 19.8–21.7 μL/mL [39]. For L929 cells, betaine-based systems with polyols/sugars have shown EC_50_ values on the order of 1–2 M [40]. In the same cell line, low cytotoxicity has been reported for ChCl-based DES at 25 mg/mL, with lower viability observed for formulations using an acid as the HBD [41].

In HaCaT cells, some DES have been reported to increase cell viability above the control; notably, ethylene glycol evaluated alone increased viability by up to 40% (e.g., at 50 μg/mL) [42]. In this work, the betaine:ethylene glycol:water (1:3:5) (DES 3) extract maintained viability close to or above 100% in HaCaT cells, and at higher concentrations than other DES tested in the literature. For example, Costa et al. [36] reported, for the same cell line, DES extracts based on proline and polyols with viabilities above 50% at concentrations below 50 μg/mL, with a marked decrease when the concentration increased to 100 μg/mL.

### 3.3. Stability Study

**Figure 2:**
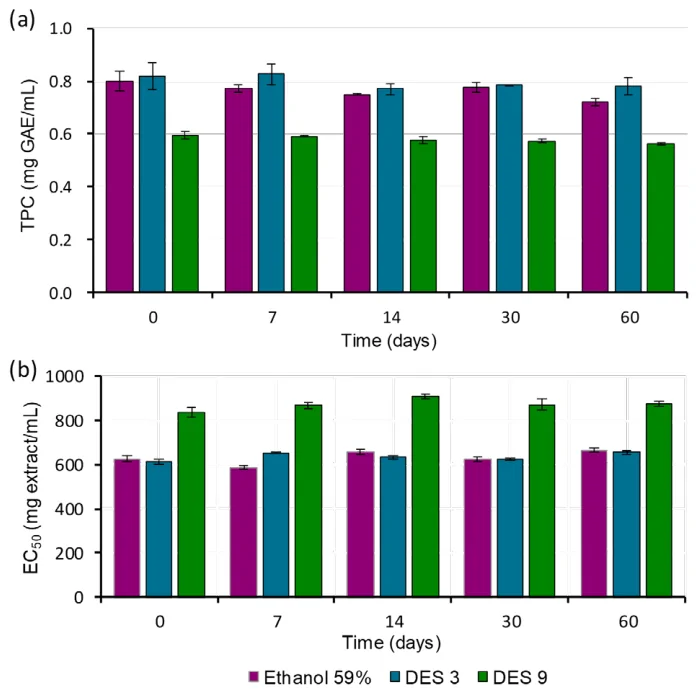
Stability assays under room-temperature storage: (**a**) Total phenolic content (TPC) in terms of mg gallic acid equivalents (GAE)/mL and (**b**) antioxidant activity expressed as EC_50_. The statistical analysis for the data presented in this figure is available in the Supporting Information (Table S1).

**Figure 3:**
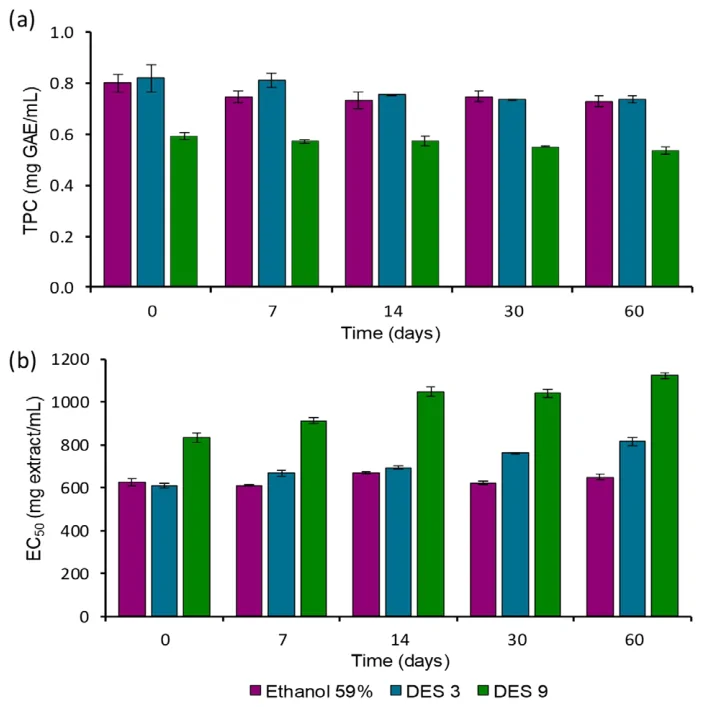
Stability assays at 40 °C under dark storage: (**a**) Total phenolic content (TPC) in terms of mg gallic acid equivalents (GAE)/mL and (**b**) antioxidant activity expressed as EC_50_. The statistical analysis (ANOVA followed by Dunnett’s test) for the data presented in this figure is available in the Supporting Information (Table S2).

Storage stability is a key factor, as the extraction solvent can influence the preservation of the bioactive properties. Therefore, the TPC and the antioxidant activity (EC_50_) were monitored for 60 days under two conditions: room temperature with exposure to natural light, and 40 °C in the dark for the ethanol/water extract and the DES 3 and DES 9 extracts. At room temperature (Figure 2), the ethanolic extract and DES 9 extract exhibited a slight decrease in the TPC over 60 days, with a significant decrease of 10.1% and 5.2% (*p* < 0.05), respectively. Alternatively, the DES 3 extract remained more stable with a decrease of 4.6% (*p* > 0.05) (Figure 2a). For the antioxidant activity assay, this effect was also slight but statistically significant for all the three extracts (*p* < 0.05); i.e., the EC_50_ showed an increase of 6.2%, 7.4%, and 5.0% for the ethanolic extract, and the DES 3, and DES 9 extracts, respectively (Figure 2b). At 40 °C (Figure 3), the TPC decreased significantly in the ethanol/water extract and the two DES extracts (*p* < 0.05), with the following decrease at the end of the assay: ethanol/water (8.9%), DES 3 (9.9%), and DES 9 (9.6%) (Figure 3a). The EC_50_ varied again significantly for all three extracts (*p* < 0.05), with a larger increase for DES 9 (34.4%) and DES 3 (33.8%) compared with the ethanol/water extract (3.9%) (Figure 3b), indicating a more pronounced loss of antioxidant activity at this temperature.

The TPC and EC_50_ results suggest that the antioxidant activity is not solely determined by the TPC, but also by changes in the qualitative profile of the extracted compounds, as more pronounced variations were observed in the EC_50_ at 40 °C. This means that at this temperature, the greater increase in EC_50_ together with a slighter decrease in TPC suggests a more substantial loss of activity due to degradation and/or transformation of thermolabile compounds. These results are consistent with those reported by Esparza et al. [43] for 50% ethanolic extracts of VS, where both the TPC and the antioxidant activity decreased over time. These authors further reported that stilbenes (*trans*-resveratrol and *trans*-ε-viniferin) are highly affected by light exposure than other phenolic compounds, besides presenting thermal susceptibility.

Nonetheless, all three extracts showed a limited variation over 60 days, indicating good overall stability within the evaluated storage period; the DES 3 extract exhibited the highest stability at room temperature with light, whereas the hydroethanolic extract showed the best stability at 40 °C in the dark. The literature suggests that DES could protect bioactive compounds through solute-solvent interactions (e.g., hydrogen bonds), increased viscosity, and microstructural effects. However, these considerations are included as possible mechanisms that depend on the DES composition, the HBA:HBD ratio, and the water content [44,45].

Concerning the comparison of the DES and ethanol:water extracts, the present results contrast with Costa et al. [36], who reported lower stability for spent coffee grounds extracts obtained with ethanol and higher stability for extracts obtained with L-proline-based DES at both room temperature and 40 °C. In another study using orange peel extracts, the DES lactic acid:glucose and L-proline:malic acid showed temperature-dependent stability: the first was more stable at 4 °C and the latter at 25 °C, with both outperforming ethanol in terms of TPC stability [13]. Overall, these results support that selection of the extraction medium and vehicle should consider not only extraction yield, but also the antioxidant activity, and the intended storage conditions, since the impact on both seems to depend on the phenolic source and, therefore, on the type of phenolic compound.

### 3.4. Influence of the Factors on DES Extraction from Vine Shoots

The extraction efficiency of bioactive compounds from lignocellulosic biomass depends on the interaction between the plant matrix, the solvent, and the extraction conditions. The use of DES as a solvent, combined with controlled heating, can modify DES properties [30] and enhance solubilisation and mass transfer of compounds into the extraction medium [46]. Based on the previous results (extractive yield, cytotoxicity profile, and stability), betaine-ethylene glycol-water (1:3:5) (DES 3) was selected as the most promising system to proceed to the optimisation of VS extraction. This was done using a Box–Behnken experimental design, which enables us to evaluate the effects of temperature (°C), extraction time (min), and (L/S) on both the TPC and the antioxidant activity.

Table 3 presents the experimental conditions for this extraction and the corresponding results for the resulting extracts. The TPC was reported as mg GAE/mL (extract concentration), relevant for the direct use of the DES extract, and as mg GAE/g dry VS to compare recovery yield across conditions. The antioxidant activity was reported as EC_50_ (mg extract/mL). The TPC (mg GAE/mL) ranged from 0.61 (run 2: 40 °C, 120 min, L/S 30:1) to 2.25 (run 5: 80 °C, 120 min, L/S 10:1), in agreement with EC_50_ values, which ranged from 791.8 (run 2) to 158.1 mg extract/mL (run 5). TPC on a dry-biomass basis (mg GAE/g dry VS) ranged from 20.6 (run 16: 40 °C, 60 min, L/S 20:1) to 36.0 (run 12: 80 °C, 120 min, L/S 30:1). The central point replicates (runs 1, 7, 8, 10, and 17; 60 °C, 120 min, L/S 20:1) showed low variability: TPC ranged from 1.03 to 1.08 mg GAE/mL, biomass-referred TPC from 26.5 to 27.7 mg GAE/g dry VS, and EC_50_ from 417.8 to 447.8 mg extract/mL. This consistency indicates good experimental repeatability and supports the adequacy of the model for optimisation.

The L/S ratio had a considerable influence on extract concentration and biomass-referred TPC. At higher solid loading (L/S 10:1), more concentrated extracts with better antioxidant activity were obtained; for example, run 5 (80 °C, 120 min) showed the highest TPC (2.25 mg GAE/mL) and the lowest EC_50_ (158.1 mg extract/mL). In contrast, reducing the solid loading (L/S 30:1) increased biomass-referred TPC, as observed in run 12 (80 °C, 120 min) with 36.0 mg GAE/g dry VS, but with a lower extract concentration (0.93 mg GAE/mL) and a higher EC_50_ (477.0 mg extract/mL). These results show that a higher recovery per unit biomass does not necessarily translate into greater antioxidant activity of the extract, which is particularly relevant when the DES extract is intended for direct use, as in this work. This behaviour has also been described in the literature, where the effect of the L/S ratio is reported to depend on the study objective and the plant matrix. In VS, a L/S ratio of 10:1 has been reported to maximise r-viniferin extraction compared with 5:1. However, increasing the ratio beyond 20:1 reduces its concentration [20]. Silva et al. [47], working with orange and coffee residues, observed higher phenolic concentrations (mg GAE/L) at moderate L/S ratios (10:1–15:1) than at higher ratios. This trend can be explained by the fact that moderate ratios maintain a favourable concentration gradient and prevent solvent saturation, whereas excessive increases in DES volume tend to dilute the extract (reducing TPC). In contrast, other studies have reported higher TPC values (mg GAE/g) at higher L/S ratios in *L. japonica* (15:1) and wild thyme (30:1), reinforcing that selection of the L/S ratio should be aligned with the intended end use of the extract [17,48].

Temperature is a crucial factor affecting the extractive capacity of DES and the antioxidant activity of the resulting extracts. Increasing temperature during extraction reduces the characteristic viscosity of these solvents by weakening their hydrogen-bonding networks, thereby enhancing mass transfer and solvent diffusion into the plant matrix and promoting the solubilisation of phenolic compounds [49]. This effect is clearly evidenced when comparing 40 and 80 °C under equivalent time and L/S conditions (60 min and 20:1). Run 16 at 40 °C showed a TPC of 0.81 mg GAE/mL and an EC_50_ of 658.2 mg extract/mL, whereas Run 3 at 80 °C increased TPC to 1.31 mg GAE/mL and reduced EC_50_ to 345.3 mg extract/mL, corresponding to an ≈62% increase in TPC and an ≈47% decrease in EC_50_, indicating improved extract concentration and antioxidant activity. A similar pattern was observed at 180 min with the same L/S ratio (20:1), where run 9 (40 °C) yielded a TPC of 1.02 mg GAE/mL and an EC_50_ of 465.6 mg extract/mL, compared with 1.28 mg GAE/mL and an EC_50_ of 345.0 mg extract/mL at 80 °C (run 6), representing a 25.5% increase in TPC and a 25.9% reduction in EC_50_. This suggests that the impact of temperature becomes less pronounced at longer extraction times. Consistently, biomass-referred TPC also increased with temperature, from 20.6 to 33.4 mg GAE/g dry VS at 60 min and from 26.1 to 32.7 mg GAE/g dry VS at 180 min (L/S 20:1), confirming improved recovery per unit of raw material. Studies on orange and coffee by-products extracted with DES have shown that increasing temperature from 40 °C to 80 °C (120 min) significantly increases TPC, whereas other studies in wild thyme and *L. japonica* flowers reported maximum phenolic recovery and higher antioxidant activity at 60 °C (10 min) and 75 °C (180 min), respectively [17,47,48]. Similarly, studies on VS identified an optimal temperature for resveratrol extraction of 85 °C (30 min), while higher temperatures led to losses attributed to thermal degradation [24]. In contrast, the extraction from VS by microwave-assisted extraction with 58% ethanol, showed that TPC, resveratrol, and viniferin can be maximised at more severe but very short conditions (155 °C, 3.5 min). It is suggested that exposure time influences degradation processes [19].

Regarding the extraction time, its effect depended on temperature. At 80 °C and an L/S ratio of 20:1, extending the extraction from 60 min (run 3) to 180 min (run 6) produced no relevant changes: TPC decreased slightly from 1.31 to 1.28 mg GAE/mL (33.4 to 32.7 mg GAE/g dry VS) and EC_50_ remained essentially constant (345.3 to 345.0 mg extract/mL), suggesting that the process approached equilibrium and that extraction occurs predominantly during the early stages. In contrast, at 40 °C and L/S of 20:1, increasing the time from 60 min (Run 16) to 180 min (Run 9) increased the TPC from 0.81 to 1.02 mg GAE/mL, reduced the EC_50_ from 658.2 to 465.6 mg extract/mL, and raised the biomass-referred TPC from 20.6 to 26.1 mg GAE/g dry VS, corresponding to an ≈26% increase in TPC and an ≈29% decrease in EC_50_. This indicates that, under less severe conditions, longer extraction times are required to enhance the recovery of phenolic compounds, but it does not reach the values obtained at 80 °C. Similar behaviour has been described in other optimisation studies, where short times may be insufficient to extract bioactive compounds, whereas prolonged times do not improve yield and may even promote degradation [13]. For wild thyme extracts obtained with DES, the effect of time was more moderate; at a constant L/S ratio, extending the extraction from 60 to 180 min increased TPC by approximately 1.9% at 40 °C and 5.5% at 70 °C, with the highest phenolic content obtained at 180 min [17]. In limonene extractions, yield increased from 30 to 150 min and then stabilised (150–180 min), suggesting an approach to diffusion equilibrium (Fick’s second law); moreover, prolonged times may favour degradation [50]. In grapevine roots, ultrasound-assisted extraction of stilbenes using ChCl-based DES showed a maximum at 10 min, whereas longer times reduced yield, which was attributed to energy-induced polymerisation [20].

### 3.5. Optimisation of DES Extraction and Characterisation from Vine Shoots

Using RSM, second-order polynomial models were fitted to the evaluated responses: TPC (mg GAE/mL), biomass-referred TPC (mg GAE/g dry VS), and EC_50_ (mg extract/mL). The models, expressed in terms of coded factors, are presented in Equations (3)–(5) as functions of temperature (A), extraction time (B), and L/S ratio (C). Model adequacy was assessed by ANOVA. In all three cases, the models were statistically significant (*p* < 0.05) and showed good fit (R^2^ ≥ 0.98 and adjusted R^2^ ≥ 0.95), together with low experimental dispersion (CV = 2.60–3.63%). Lack of fit was not significant (*p* > 0.05), indicating that the models adequately described the responses within the experimental domain studied.

TPC (mg GAE/mL) = 1.06 + 0.17·A + 0.028·B − 0.64·C − 0.061·AB + 0.077·A^2^ − 0.035·B^2^ + 0.29·C^2^ (3)

CV = 2.60%; R^2^ = 0.9978; Adj. R^2^ = 0.9959
TPC (mg GAE/g) = 27.28 + 4.17·A + 1.21·B + 1.94·C − 1.50·AB + 1.47·AC
− 0.88·BC + 2.56·A^2^ − 1.57·B^2^ − 0.91·C^2^ (4)

CV = 2.91%; R^2^ = 0.9804; Adj. R^2^ = 0.9511
EC_50_ (mg extract/mL) = 435.60 − 98.65·A − 47.12·B + 226.78·C + 48.08·AB
− 68.45·AC − 45.21·BC − 2.17·A^2^ + 20.08·B^2^ − 26.91·C^2^ (5)

CV = 3.63%; R^2^ = 0.9973; Adj. R^2^ = 0.9933

**Figure 4:**
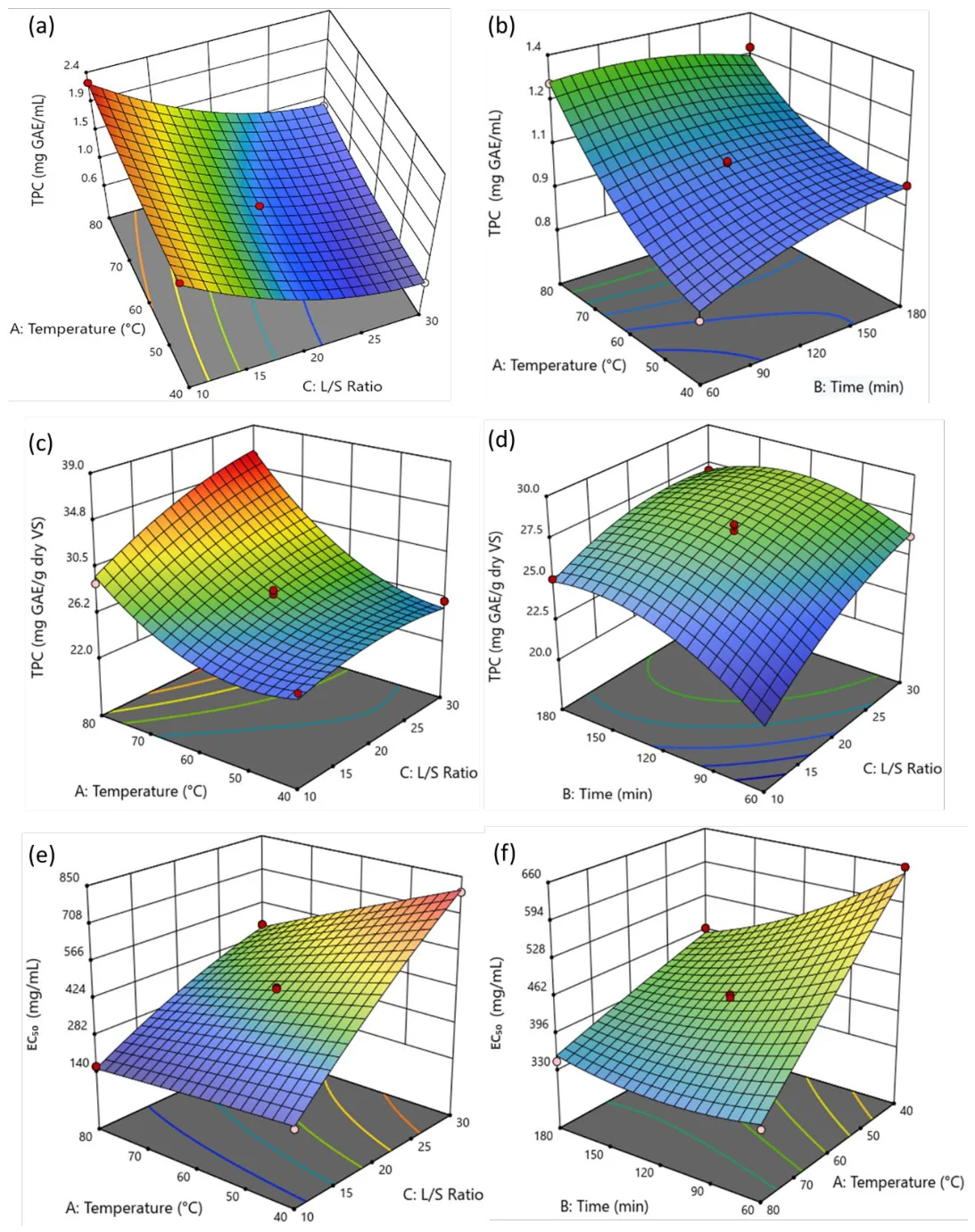
Response surface plots for vine shoots (VS) extraction using DES 3: (**a**,**b**) total phenolic content (TPC) (mg gallic acid equivalents (GAE)/mL), (**c**,**d**) biomass-referred TPC (mg GAE/g dry VS), and (**e**,**f**) EC_50_ (mg extract/mL). In each plot, the third factor was fixed at its central level: 60 °C, 120 min, or liquid-to-solid (L/S) ratio 20:1. The colour gradient represents the magnitude of the responses, with the lowest values indicated in blue and the highest values in red.

According to the coefficients of the aforementioned equations, temperature and the L/S ratio were the most significant factors for all responses (*p* < 0.05), whereas time showed a more interaction-dependent effect. For the TPC (mg GAE/mL) (Equation (3)), temperature and time had positive effects, while the L/S ratio had a negative effect, indicating that increasing the L/S ratio lowers the phenolic concentration of the extract. This is reflected in the response surface plots (Figure 4a), where the highest TPC values occur at higher temperatures and a lower L/S ratio (10:1). As shown in Figure 4b, the influence of the time extraction was lower, although increasing extraction time at high temperature may negatively affect phenolic concentration, with maximum values observed at intermediate times. For biomass-referred TPC (mg GAE/g dry VS) (Equation (4)), the three factors (linear terms) showed a positive influence, although temperature was the most significant factor to increase the TPC. A positive interaction between temperature and L/S ratio was observed, indicating that increasing both factors simultaneously enhanced the phenolic yield (Figure 4c). In contrast, the temperature-time and time-L/S ratio interactions showed negative influences, evidenced by a decrease in TPC.

Moreover, as shown in Figure 4d, the highest TPC (mg GAE/g dry VS) was obtained at intermediate times and intermediate-to-high L/S ratios. For EC_50_ (mg extract/mL) (Equation (5)), temperature and time showed negative effects, indicating that increasing these factors decreases EC_50_ and improves antioxidant activity. In line with the concentration of phenolic compounds, the L/S ratio showed a positive effect, associated with increased EC_50_ and, therefore, a lower antioxidant activity. Figure 4e shows that, at low solid loading (L/S of 30:1), increasing temperature promotes a decrease in EC_50_. In addition, at high temperatures, the effect of the extraction time was negligible (Figure 4f).

The response surface plots and models indicate that maximising biomass-referred TPC (mg GAE/g dry VS) is associated with higher L/S ratios (30:1), whereas maximising phenolic concentration and extract activity (higher TPC, mg GAE/mL, and lower EC_50_) is favoured by lower L/S ratios (10:1). This agrees with Pavlić et al. [17], who, when optimising the extraction of wild thyme, found temperature to be the dominant factor affecting TPC and antioxidant activity, followed by the L/S ratio, whereas time had a smaller influence. Similarly, Makarova et al. [48] reported that for *L. japonica* extracted with DES, the L/S ratio was the dominant factor, followed by the temperature and a smaller effect of time.

Considering that one of the main advantages of DES is its potential direct application in cosmetic, food, or pharmaceutical formulations, the extract concentration and its antioxidant activity were targeted, maximising the TPC (mg GAE/mL) and minimising the EC_50_ (mg extract/mL), since these determine the capacity of the extracts when incorporated into the final matrix. However, considering that VS were evaluated on a dry basis, biomass-referred TPC (mg GAE/g dry VS) was also included in the optimisation of the extraction as an indicator of recovery efficiency, relevant for the better use of the biomass. Therefore, a multiple-response optimisation using the desirability function was applied via Design-Expert software to simultaneously maximise both the TPC in terms of concentration (mg GAE/mL) and the biomass-referred TPC (mg GAE/g dry VS), while minimising the EC_50_ (mg extract/mL). This mathematical tool helps find the best compromise between the studied responses. According to the experimental design, the optimal extraction conditions predicted by the global model were 80 °C, 127 min, and a L/S ratio of 10:1. Under these conditions, the model predicted TPC values of 2.2 mg GAE/mL and 29.7 mg GAE/g dry VS, and EC_50_ of 155 mg extract/mL. To validate the model, these conditions were reproduced experimentally in triplicate, obtaining a TPC of 2.35 ± 0.05 mg GAE/mL and 30.3 ± 0.6 mg GAE/g dry VS, and an EC_50_ of 162 ± 6 mg extract/mL, with errors relative to the predicted values of 5.1%, 1.9%, and 4.6%, respectively. In addition, the antioxidant activity of the optimised DES 3 extract was also measured using the FRAP and ABTS assays, obtaining 11.4 ± 0.1 mg TE/g dry VS and 77.7 ± 2.9 mg TE/g dry VS, respectively. These values were higher than those for 59% ethanol (8.0 mg TE/or 54.1 mg TE/g dry VS), the original DES 3 extract (8.6 mg TE/g dry VS and 56.7 mg TE/g dry VS), and the DES 9 extract (7.1 mg TE/g dry VS and 42.4 mg TE/g dry VS).

Moreover, *trans*-resveratrol and *trans-ε*-viniferin were quantified in the extract obtained under optimal conditions using HPLC-UV, as both are highly relevant VS compounds to formulating eco-friendly ingredients in the cosmetic industry [51]. The concentration measured was 16 ± 2 µg/mL (200 ± 2 µg/g dry VS) and 31 ± 3 µg/mL (400 ± 40 µg/g dry VS), respectively. Although DES can enhance the extraction of phenolic compounds, their affinity for individual molecules largely depends on the chemical nature of the target compound, solvent polarity, and the interactions established between the DES hydrogen-bonding network and the solute. Several authors have reported that small variations in DES composition, including the HBA:HBD ratio and water content, can significantly modify the ability to extract specific compounds [49]. In ultrasound-assisted VS extracts, resveratrol concentrations of 0.52–2.47 mg/g and ε-viniferin concentrations of 0.07–0.78 mg/g have been reported using different DES. In that study, the most effective system was ChCl:1,2-propanediol (1:2, mol/mol), with values increasing after optimisation of the extraction process, whereas glucose:urea (3:1, mol/mol) showed the lowest yield [20]. Other studies on VS from different cultivars, optimised using ChCl–ethylene glycol and microwave-assisted heating, reported 0.90–6.1 mg resveratrol/g and 0.7–1.5 mg ε-viniferin/g. The observed variations have been attributed to differences in extraction methods, DES composition, and the nature of the plant material [24].

Additionally, the obtained DES extract was characterised by HPLC-QTOF-MS and MS/MS to provide more information on its phenolic composition. Table 4 shows the retention time, the mass-to-charge ratio (*m*/*z*) of the deprotonated ions, and the molecular formula of the neutral form. All the compounds showed errors lower than 5 ppm, and the score was higher than 90% (Table S3). The *m*/*z* data of the mass fragments are shown in Table S3. In addition to *trans*-resveratrol and its dimer, 10 stilbenes were found, along with 7 phenolic acids and their derivatives, 2 phenolic aldehydes, and 4 flavan-3-ols (Table 4). The phenolic qualitative profile and fragmentation pattern (Table 4, Figure S2, and Table S3) were consistent with previous studies on this cultivar using aqueous ethanolic solutions, but the *trans*-ε-viniferin to *trans*-resveratrol ratio varied depending on how the extraction was performed on VS [2,19]. Other additional minor phenolic compounds found in the studied DES extract were glucogallin, dihydroxybenzoic acid hexoside, and *p*-coumaric acid glucoside, which have been characterised in previous studies on VS and/or red grapes [52,53].

### 3.6. Cytotoxicity of the Optimal DES Extract

**Figure 5:**
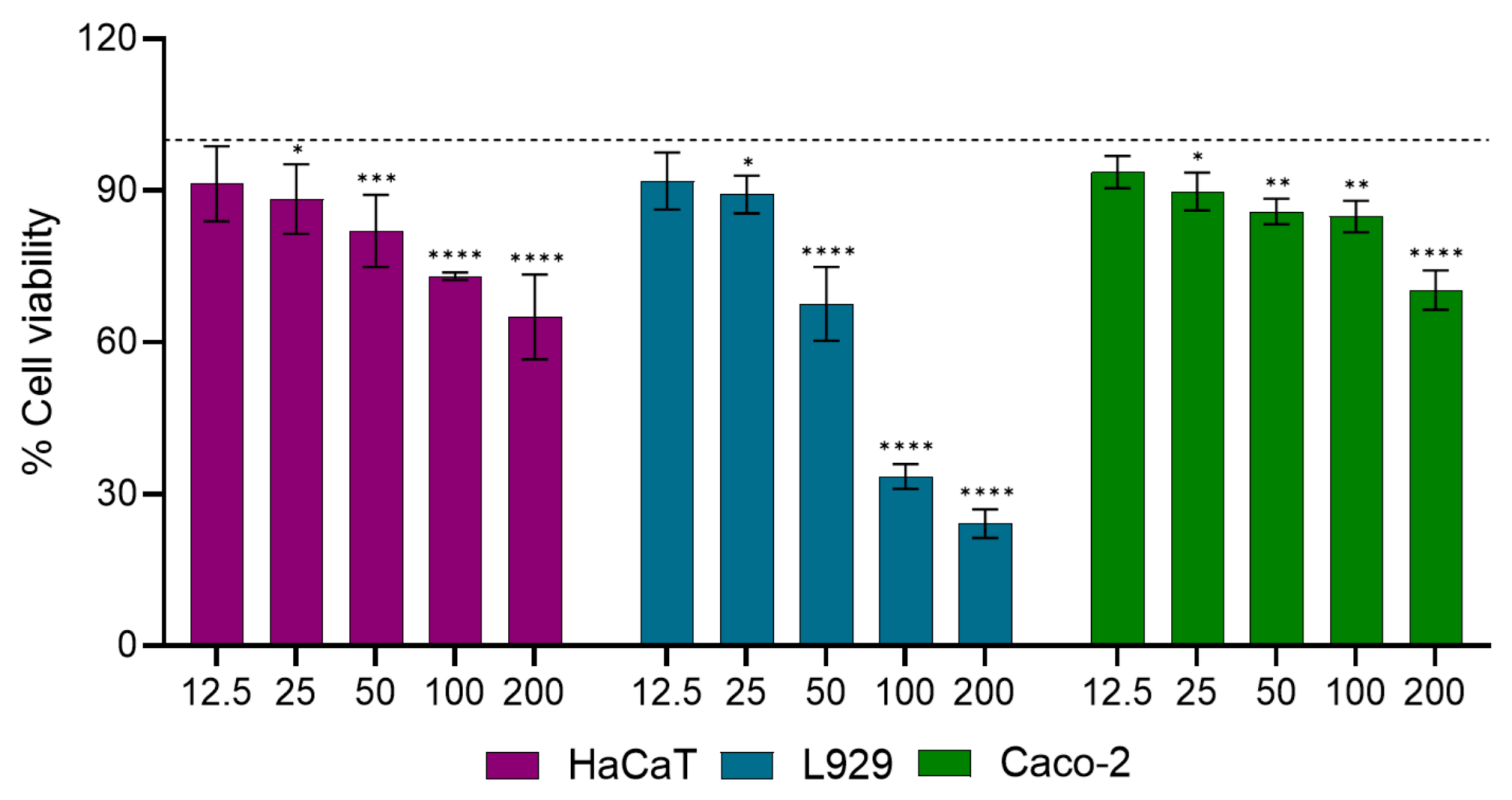
Cell viability after 24 h exposure to the extract obtained using DES 3 under optimal extraction conditions (80 °C, 127 min, and L/S 10:1). Assays in HaCaT, L929, and Caco-2 cells (12.5–200 mg extract/mL). The dashed line represents 100% viability (negative control). The means have been compared by two-way ANOVA followed by Dunnett’s test with the negative control (* *p* < 0.05, ** *p* < 0.01, *** *p* < 0.001, **** *p* < 0.0001).

Compared to the extraction with DES 3 under initial conditions (40 °C, 60 min, L/S 20:1), the optimisation increased the extraction yield (TPC) and the antioxidant properties (about 3 and 4 times, respectively). Therefore, cytotoxicity was re-evaluated in the three cell lines under the same conditions for the optimised DES extract (Figure 5). In HaCaT, a potentially cytotoxic effect was observed only at the highest concentration, with viabilities above 70% up to 100 mg extract/mL (73.0%) and a decrease to 65.0% at 200 mg extract/mL (*p* < 0.05). L929 showed the greatest susceptibility, with viabilities below 70% from 50 mg extract/mL (67.6%), whereas at 12.5 and 25 mg extract/mL they remained above 89% (*p* < 0.05). For Caco-2, the model showing the highest tolerance to the extract, viabilities remained above 84% up to 100 mg/mL and decreased to 70.3% at 200 mg extract/mL (*p* < 0.05), a value close to the established limit (EN ISO 10993-5) [36]. These results suggest that cytotoxicity could increase under more demanding extraction conditions driven by its higher TPC, potential changes in the compositional profile, or osmotic effects associated with a higher total solute load. Therefore, it is important to re-evaluate biocompatibility not only on the DES system, but also following any optimisation of the extraction of natural compounds.

Notably, the DES 3 extract exhibited low cytotoxicity levels—especially in HaCaT and Caco-2 cells—despite being tested at very high concentrations compared to other DES extracts. For example, a previous study evaluated ChCl-based DES extracts of *Curcuma longa* on HaCaT cells at 0.125 mg/mL, resulting in a decrease in cell viability exceeding 50% in some cases, depending on the DES system [54]. ChCl-based DES extract from jambolan fruit showed no cytotoxicity up to 12.5 mg/mL on HaCaT cells, but a remarkable cytotoxic effect was found using higher concentrations (e.g., 25–100 mg/mL) [55].

### 3.7. Limitations of the Study

DES are frequently praised as green, non-toxic alternatives to conventional industrial solvents. However, when ethylene glycol is introduced as the HBD, the green label requires a critical look; for example, compared with sorbitol, which has a well-established safety profile [56]. While ethylene glycol is only poorly absorbed through the dermal route, ingested ethylene glycol is absorbed rapidly, exhibiting high toxicity both directly and through its metabolites [57]. Its systemic toxicity is primarily driven by its metabolism, metabolising into glycolic acids and oxalates. While ethylene glycol can result in these metabolites, their formation rate depends on the animal model [58]. When applied in DES, the DES 3 extract had better biocompatibility in the studied cells. Interestingly, previous studies on the cytotoxicity profile of DES suggest that DES are not simply an additive sum of their starting components and the cells’ responses are not easily explained [42]. For instance, these authors observed contrasting results when changing the HBA using ethylene glycol as HBD, which shifted the outcome from cytotoxic effects to increased cell viability. ChCl:ethylene glycol has shown lower toxicity towards multicellular organisms, such as fish, hydra, fungi, and garlic, compared to its individual components [59,60]. This effect has been attributed to the strong hydrogen bonding network of the DES components that reduces their bioavailability [61]. Using an in silico approach, HBDs were classified according to their environmental toxicity; among these, alcohol-based DES, including ethylene glycol, were identified as the lower-toxicity contributors [62]. Intriguingly, its polymer, polyethene glycol, has a good safety profile, being used in pharmaceuticals, cosmetics, and food products [63]. Previous works have shown the ability of DES combining ChCl:ethylene glycol (1:2, 40% water), ChCl:ethylene glycol (1:5 molar ratio, 30% water), betaine:ethylene glycol (1:3), and tetrabutylammonium chloride:dichloroacetic acid:ethylenglycol (4:2:1, 30% water) to extract phenolic compounds from other plant matrices [61,64,65,66]. These studies suggested that DES alters the biomass surface to release phenolic compounds, and its components offer different interaction modes with these compounds that favour extraction; ethylene glycol provides hydroxyl groups, and water promotes solubilisation and mass transfer [61,64].

Overall, the data are only a preliminary indication of the safety of the studied betaine-based DES extract–only based on the studied cells–and would still require further toxicological and regulatory assessment to be used for ready-to-use applications. Although cell viability assays are useful, they do not demonstrate systemic safety, metabolism, or regulatory acceptability for real applications. For example, in topical formulations, its use to formulate ingredients seems interesting, but the use of betaine-based DES or other DES with ethylene glycol is problematic and requires further research—particularly in vivo studies—to validate their antioxidant effects and toxicological profiles, including their metabolism. The present work provides other combinations that are more suitable with respect to the safety of the individual components, such as DES 9, based on GRAS-approved components [67], despite lower TPC and antioxidant activity. Future research should focus on validating these comparative findings in vivo. The use of betaine-based DES to extract phenolic compounds may offer an alternative to conventional organic solvents, which require further purification steps, to align the process with the principles of the circular bioeconomy in the food sector. However, safety evaluations of DES and DES extracts across food, pharmaceutical, and topical applications are still limited, and more work is needed.

The utility of betaine-based DES extracts could extend beyond human consumption or topical applications, such as in materials, as ethylene glycol is applied in antifreeze, coolants, chemical processing, etc. [58]. For example, the work by Basar et al. showed that the antioxidant properties of films can be enhanced by combining ChCl:ethylene glycol to solubilise resveratrol and electrospinning [68].

## 4. Conclusions

The betaine-based DES proved to be an efficient solvent to recover phenolic compounds with antioxidant activity, measured by the DPPH assay, from VS. The screening of 14 formulations suggested that both the HBD and the water content have an important influence on the extraction yield. This screening led to the selection of the betaine-ethylene glycol-water (1:3:5) system, as it showed a yield comparable to the control ethanolic extract, along with favourable stability and improved biocompatibility. Using RSM, optimal conditions were determined for VS extraction with that DES system at 80 °C, 127 min, and a L/S ratio of 10:1. These conditions generated an extract with a phenolic content of 2.35 mg GAE/mL (30.3 mg GAE/g dry VS), an antioxidant activity with EC_50_ of 162 mg/mL, and with the presence of *trans*-resveratrol and *trans-ε*-viniferin. However, the increase in phenolic compound concentration after optimisation, although relatively low, intensified the cytotoxic response depending on the cell line and dose. Overall, these results provide preliminary support for the use of betaine-based DES to extract phenolic compounds from VS, including *trans*-resveratrol and its dimer, and obtain extracts with antioxidant activity. Nonetheless, the studied DES extracts should be targeted in future metabolomic and in vivo studies to enable a deeper understanding of their antioxidant activity, systemic effects and the metabolites generated. Their toxicological properties should also be better established in vivo for real ready-to-use applications. Moreover, future studies should focus on applying green intensification technologies, such as ultrasound or microwave, to favour more energetically efficient extraction.

## Figures and Tables

**Table 1:** Composition and molar ratios of the DES used in this study.

DES No.	Composition	Molar Ratio	Density (g/cm^3^)
1	Betaine:ethylene glycol	1:3	1.100
2	Betaine:ethylene glycol:water	1:3:1	1.117
3	Betaine:ethylene glycol:water	1:3:5	1.146
4	Betaine: ethylene glycol:water	1:3:10	1.167
5	Betaine:1,2-propanediol	1:3	1.104
6	Betaine:1,2-propanediol:water	1:3:1	1.083
7	Betaine:lactic acid	1:2	1.410
8	Betaine:lactic acid:water	1:2:1	1.200
9	Betaine:sorbitol:water	3:1:10	1.168
10	Betaine:sorbitol:water	1:1:3	1.228
11	Betaine:sorbitol:water	1:1:5	1.241
12	Betaine:glycerol	1:2	1.168
13	Betaine:glycerol:water	1:2:1	1.175
14	Betaine:glucose:water	5:2:10	1.165

**Table 2:** Total phenolic content (TPC) and antioxidant activity (EC_50_) of extracts obtained with different deep eutectic solvent (DES) systems and the control ethanolic extract (59% *v*/*v*, ethanol/water).

DES No.	TPC (mg GAE/mL)	TPC (mg GAE/g dry VS)	EC_50_ (mg extract/mL)	FRAP (mg TE/g dry VS)	ABTS (mg TE/g dry VS)
1	0.62 ± 0.01	14.1 ± 0.2	709.5 ± 11.3	6.7 ± 0.1	43.6 ± 1.6
2	0.73 ± 0.02	16.6 ± 0.5	747.0 ± 10.5	6.1 ± 0.1	45.9 ± 0.4
3	0.82 ± 0.05	18.9 ± 1.2	611.2 ± 11.3	8.6 ± 0.1	56.7 ± 2.6
4	0.81 ± 0.03	18.6 ± 0.6	625.9 ± 17.3	8.2 ± 0.5	52.7 ± 3.7
5	0.44 ± 0.02	9.9 ± 0.4	1162.5 ± 30.6	3.9 ± 0.2	25.3 ± 0.4
6	0.44 ± 0.02	9.8 ± 0.4	1029.5 ± 13.5	4.4 ± 0.1	25.5 ± 2.7
7	0.54 ± 0.03	12.6 ± 0.6	1307.5 ± 17.6	5.7 ± 0.2	17.0 ± 0.9
8	0.78 ± 0.01	17.8 ± 0.2	955.6 ± 28.9	6.2 ± 0.1	20.5 ± 1.7
9	0.59 ± 0.01	13.6 ± 0.3	835.3 ± 21.8	7.1 ± 0.5	42.4 ± 0.8
10	0.56 ± 0.02	12.7 ± 0.4	929.0 ± 22.3	6.5 ± 0.2	32.7 ± 1.8
11	0.51 ± 0.01	11.8 ± 0.2	871.8 ± 4.1	7.0 ± 0.2	33.4 ± 1.8
12	0.41 ± 0.01	9.4 ± 0.1	1522.1 ± 93.3	3.8 ± 0.1	24.3 ± 2.4
13	0.46 ± 0.02	10.3 ± 0.5	1151.8 ± 15.2	4.7 ± 0.2	27.3 ± 1.5
14	0.55 ± 0.02	12.6 ± 0.5	1344.2 ± 25.6	5.0 ± 0.2	29.3 ± 0.4
Control	0.80 ± 0.04	16.4 ± 0.7	625.8 ± 14.4	8.0 ± 0.1	54.1 ± 1.7

GAE: gallic acid equivalents; VS: vine shoots.

**Table 3:** Experimental conditions and results of TPC and antioxidant activity (EC_50_) for vine shoots (VS) extraction using DES 3 (betaine:ethylene glycol:water, 1:3:5).

Run	Temp (°C)	Time (min)	L/S Ratio	TPC ^a^ (mg GAE/mL)	TPC ^b^ (mg GAE/g)	EC_50_ (mg/mL)
1	60	120	20:1	1.07	27.3	417.8
2	40	120	30:1	0.61	25.9	791.8
3	80	60	20:1	1.31	33.4	345.3
4	60	60	30:1	0.68	26.3	745.6
5	80	120	10:1	2.25	29.0	158.1
6	80	180	20:1	1.28	32.7	345.0
7	60	120	20:1	1.08	27.7	440.3
8	60	120	20:1	1.07	27.7	444.8
9	40	180	20:1	1.02	26.1	465.6
10	60	120	20:1	1.06	27.2	427.2
11	60	60	10:1	2.05	26.4	203.9
12	80	120	30:1	0.93	36.0	477.0
13	60	180	10:1	1.95	25.1	202.3
14	60	180	30:1	0.70	27.1	741.7
15	40	120	10:1	1.92	24.8	199.1
16	40	60	20:1	0.81	20.6	658.2
17	60	120	20:1	1.03	26.5	447.8

Temp: Temperature; L/S Ratio (mL DES/g dry VS); TPC: Total phenolic content, expressed as ^a^ mg gallic acid equivalents (GAE)/mL and ^b^ mg GAE/g dry VS; EC_50_ expressed as mg extract/mL.

**Table 4:** Phenolic compounds and other compounds characterised in the DES 3 (betaine:ethylene glycol:water, 1:3:5) extract obtained at optimal conditions (80 °C, 127 min, and a L/S ratio of 10:1).

No	Retention Time (min)	[M − H]^−^ *m*/*z*	Molecular Formula	Proposed Compound
Phenolic Compounds				
1	0.9	169.015	C_7_H_6_O_5_	Gallic acid
2	1.2	331.067	C_13_H_16_O_10_	Glucogallin (galloyl glucose)
3	1.5	315.072	C_13_H_16_O_9_	Dihydroxybenzoic acid hexoside
4	1.8	153.019	C_7_H_6_O_4_	Dihydroxybenzoic acid
5	2.7	137.024	C_7_H_6_O_3_	3,5-Dihydroxybenzaldehyde
6	4.3	121.030	C_7_H_6_O_2_	Hydroxybenzaldehyde
7	4.6	197.046	C_9_H_10_O_5_	4-hydroxy-3,5-dimethoxybenzoic acid (syringic acid)
8	4.9	325.093	C_15_H_18_O_8_	*p*-Coumaric acid glucoside
9	5.1	577.134	C_30_H_26_O_12_	(Epi)catechin B-type dimer
10	5.3	289.072	C_15_H_14_O_6_	Catechin
11	7.2	289.072	C_15_H_14_O_6_	Epicatechin
12	7.5	163.040	C_9_H_8_O_3_	*p*-Coumaric acid
13	8.7	471.144	C_28_H_24_O_7_	Oxidised resveratrol dimer
14	10.2	471.144	C_28_H_24_O_7_	Oxidised resveratrol dimer isomer
15	10.4	441.083	C_22_H_18_O_10_	(Epi)catechin gallate
16	11.2	469.129	C_28_H_22_O_7_	Ampelopsin A
17	13.2	227.071	C_14_H_12_O_3_	*trans*-Resveratrol
18	13.5	453.135	C_28_H_22_O_6_	Pallidol isomer
19	15.1	227.071	C_14_H_12_O_3_	Resveratrol isomer 1
20	17.3	905.259	C_56_H_42_O_12_	Hopeaphenol isomer
21	17.6	453.134	C_28_H_22_O_6_	Viniferin isomer 1
22	18.5	453.134	C_28_H_22_O_6_	*trans*-ε-viniferin
23	19.5	679.197	C_42_H_32_O_9_	Miyabenol C isomer
Other compounds				
1’	0.6	191.019	C_6_H_8_O_7_	Citric acid
2’	3.0	175.061	C_7_H_12_O_5_	2-Isopropylmalic acid
3’	5.6	373.151	C_17_H_26_O_9_	7-deoxyloganin
4’	6.0	431.192	C_19_H_30_O_8_	Vomifoliol hexoside (formate adduct)
5’	7.4	373.151	C_17_H_26_O_9_	Wine lactone hexoside
6’	11.0	187.098	C_9_H_16_O_4_	Azelaic acid

## Data Availability

The data are contained within the article or Supplementary Materials.

## Supplementary Materials

The following supporting information can be downloaded at: https://www.mdpi.com/article/10.3390/foods15203708/s1, Table S1. Statistical analysis of stability during room-temperature storage (Figure 2). One-way ANOVA followed by Dunnett’s multiple comparisons test (vs. day 0; adjusted *p*-values). ns *p* ≥ 0.05, * *p* < 0.05, ** *p* < 0.01, *** *p* < 0.001; Table S2. Statistical analysis of stability during 40 °C storage (Figure 3). One-way ANOVA followed by Dunnett’s multiple comparisons test (vs. day 0; adjusted *p*-values). ns *p* ≥ 0.05, * *p* < 0.05, ** *p* < 0.01, *** *p* < 0.001, **** *p* < 0.0001; Table S3. Main fragments measured in the MS/MS analysis of the DES 3 (betaine:ethylene glycol:water, 1:3:5) extract obtained at optimal conditions (80 °C, 127 min, and a L/S ratio of 10:1); Figure S1. Chromatograms (280 nm) of the DES 3 (betaine:ethylene glycol:water, 1:3:5) extracts, before and after optimisation, DES 9 (betaine:sorbitol:water, 3:1:10) extract and the 59% ethanolic extract; Figure S2. Base peak chromatogram of the DES 3 (betaine:ethylene glycol:water, 1:3:5) extract obtained at optimal conditions (80 °C, 127 min, and a L/S ratio of 10:1). Table 4 shows the numbering for each compound.

## Abbreviations

The following abbreviations are used in this manuscript:

ABTS	2,2′-azino-di (3-ethylbenzothiazoline-6-sulphonic acid)
ANOVA	Analysis of variance
ChCl	Choline chloride
DPPH	2,2-diphenyl-1-picrylhydrazyl
DES	Deep eutectic solvents
EC_50_	Extract concentration required to scavenge 50% of the radicals present in the assay
FRAP	Ferric ion reducing antioxidant power
GAE	Gallic acid equivalents
GRAS	Generally recognised as safe
HBA	Hydrogen-bond acceptor
HBD	Hydrogen-bond donor
HPLC	High-performance liquid chromatography
L/S	Liquid-to-solid
MS	Mass spectrometry
MTS	3-(4,5-dimethylthiazol-2-yl)-5-(3-carboxymethoxyphenyl)-2-(4-sulfophenyl)-2H-tetrazolium
RSM	Response surface methodology
TPC	Total phenolic content
Trolox	6-hydroxy-2,5,7,8-tetramethylchroman-2-carboxylic acid
VS	Vine shoots
