# Supplementary material for: Betaine-Based Deep Eutectic Solvents for Phenolic Compounds Extraction from Vine Shoots: Screening, Optimisation, and Cytotoxicity Assessment
Source: Foods. 2026 Oct 18;15(20):3708. doi: 10.3390/foods15203708 (PMC13704195; doi:10.3390/foods15203708)
Supplement: Supplementary file 1 [file foods-15-03708-s001.zip › foods-4597784-supplementary.pdf]

Betaine-Based Deep Eutectic Solvents For Phenolic Compounds Extraction From Vine Shoots: Screening, Optimisation and Cytotoxicity Assessment

Camila C. Muñoz-Realpe[1,2], Carolina Costa[3], Ana Rita Jesus[3], María del Mar Contreras[1,2*], Inmaculada Romero[1,2], Eulogio Castro[1,2], Ana Rita C. Duarte[3], Alexandre Paiva[3*]

[1] Department of Chemical, Environmental and Materials Engineering, Universidad de Jaén, 23071 Jaén, Spain; ccmunoz@ujaen.es (C.C.M.-R.); iromero@ujaen.es (I.R.); ecastro@ujaen.es (E.C.)

[2] Institute of Biorefineries Research (I3B), Universidad de Jaén, 23071 Jaén, Spain.

[3] LAQV, REQUIMTE, Departamento de Química da Faculdade de Ciências e Tecnologia, Universidade Nova de Lisboa, 2829-516 Caparica, Portugal.; acr.costa@campus.fct.unl.pt (C.C.); ar.gameiro@fct.unl.pt (A.R.J.); ard08968@fct.unl.pt (A.R.C.D.)

* Correspondence: mcgamez@ujaen.es (M.d.M.C.); alexandre.paiva@fct.unl.pt (A.P.)

Supplementary material

Table S1. Statistical analysis of stability during room-temperature storage (Fig. 2). One-way ANOVA followed by Dunnett's multiple comparisons test (vs. day 0; adjusted p-values). ns $p \geq 0.05$, * $p < 0.05$, ** $p < 0.01$, *** $p < 0.001$.

TPC - Room temperature						
	Ethanol 59%		DES 3		DES 9	
	P value	Significant	P value	Significant	P value	Significant
0 vs. 7	0.2685	ns	0.9982	ns	0.9983	ns
0 vs. 14	0.0347	*	0.2829	ns	0.1666	ns
0 vs. 30	0.4346	ns	0.5532	ns	0.0791	ns
0 vs. 60	0.0017	**	0.4875	ns	0.0092	**
**DPPH - Room temperature**						
	Ethanol 59%		DES 3		DES 9	
	P Value	Significant	P Value	Significant	P Value	Significant
0 vs. 7	0.0069	**	0.0002	***	0.1527	ns
0 vs. 14	0.0195	*	0.0137	*	0.0019	**
0 vs. 30	0.9999	ns	0.1321	ns	0.0948	ns
0 vs. 60	0.0058	**	0.0001	***	0.0222	*

Table S2. Statistical analysis of stability during 40°C storage (Fig. 3). One-way ANOVA followed by Dunnett's multiple comparisons test (vs. day 0; adjusted p-values). ns $p \geq 0.05$, * $p < 0.05$, ** $p < 0.01$, *** $p < 0.001$, **** $p < 0.0001$.

TPC – 40 °C						
	Ethanol 59%		DES 3		DES 9	
	P value	Significant	P value	Significant	P value	Significant
0 vs. 7	0.1219	ns	0.9966	ns	0.1451	ns
0 vs. 14	0.0452	*	0.0458	*	0.1980	ns
0 vs. 30	0.1368	ns	0.0114	*	0.0066	**
0 vs. 60	0.0348	*	0.0149	*	0.0006	***
**DPPH – 40 °C**						
	Ethanol 59%		DES 3		DES 9	
	P Value	Significant	P Value	Significant	P Value	Significant
0 vs. 7	0.2598	ns	0.0007	***	0.0014	**
0 vs. 14	0.0011	**	0.0001	****	0.0001	****
0 vs. 30	0.9889	ns	0.0001	****	0.0001	****
0 vs. 60	0.0389	*	0.0001	****	0.0001	****

Table S3. Main fragments measured in the MS/MS analysis of the DES 3 (betaine:ethylene glycol:water, 1:3:5) extract obtained at optimal conditions (80 °C, 127 min, and a L/S ratio of 10:1).

No	*m/z*	Proposed compound	Fragment ions (*m/z*)	ppm	Score
Phenolic compounds					
1	169.015	Gallic acid	125.0245, 107.0136, 97.0301, 79.0195, 69.0352, 51.0249	-2.49	97.95
2	331.067	Glucogallin (galloyl glucose)	313.0678, 169. 0128, 168.0056, 149.9948, 125.0234, 97.0261,	-1.05	97.76
3	315.072	Dihydroxybenzoic acid hexoside	152.0123, 153.0169, 108.0212	0.55	99.19
4	153.019	Dihydroxybenzoic acid	109.0287, 108.0209, 92.0241, 91.0185, 81.0342, 80.256	3.18	97.90
5	137.024	3,5-Dihydroxybenzaldehyde	136.0153, 119.0117, 108.0209, 92.0260, 81.0335, 65.0031, 53.0391	0.09	99.89
6	121.030	Hydroxybenzaldehyde	119.0357, 112.9856, 96.9643, 92.0270, 85.0663, 68.9963	-0.07	99.70
7	197.046	4-hydroxy-3,5-dimethoxybenzoic acid (syringic acid)	172.0256, 154.0172, 152.0109, 137.0282, 109.0270, 108.0218, 96.0446, 59.0147, 53.8562	-1.16	97.85
8	325.093	*p*-Coumaric acid glucoside	265.9900, 187.0446, 163.0405, 119.0494	-2.9	90.20
9	577.134	(Epi)catechin B-type dimer	407.0761, 381.0957, 289.0711, 245.0803, 161.0249, 137.0231, 125.0235	1.97	95.89
10	289.072	Catechin	245.0826, 221.0822, 203.0706, 161.0597, 151.0396, 137.0236, 123.0448, 109.0292, 97.0298, 57.0359	-0.37	97.10
11	289.072	Epicatechin	247.0344, 221.0843, 212.0497, 203.0712, 160.0517, 151.0381, 125.0231, 123.0434, 121.0292, 109.0279, 97.0267	-1.5	97.12
12	163.040	*p*-Coumaric acid	119.0495, 93.0343	-2.45	94.59
13	471.144	Oxidised resveratrol dimer	349.1083, 255.0673, 121.0293	1.67	94.92
14	471.144	Oxidised resveratrol dimer isomer	349.1067, 243.0638,169.0166, 121.0294	-0.46	90.29
15	441.083	(Epi)catechin gallate	289.0703, 245.0835, 169.0144, 125.0246	-0.13	98.22
16	469.129	Ampelopsin A	451.1171, 423.1232, 375.0876, 363.0877, 345.0747, 317.0822, 281.0445, 241.0499	0.46	98.81
17	227.071	*trans*-Resveratrol	185.0591, 183.0810, 181.0651, 159.0810, 157.0656, 144.0528, 143.0493, 119.0501, 117.0337, 81.0339	1.85	98.04
18	453.135	Pallidol isomer	359.0938, 265.0499, 221.0646, 178.4661, 93.0354, 68.9975	-1.57	98.47
19	227.071	Resveratrol isomer 1	185.0597, 167.7922, 157.0655, 143.0497, 131.0518	0.27	99.22
20	905.259	Hopeaphenol isomer	811.2191, 717.1782, 451.1194, 358.0840	1.08	96.62
21	453.134	Viniferin isomer 1	435.1291, 359.0964, 347.0948, 253.0521, 225.0593, 197.0597, 93.0347	0.25	99.43
22	453.134	*trans*-ε-viniferin	435.1210, 411.1245, 385.1461, 369.1108, 359.0932, 347.0921, 333.0736, 253.0494, 225.0539, 119.0514, 93.0353	0.80	98.81
23	679.197	Miyabenol C isomer	661.1911, 585.1566, 573.1531, 531.1739, 451.1181, 345.0805, 227.0738	0.07	96.80
Other compounds					
No	*m/z*	Proposed compound	Fragment ions (*m/z*)	ppm	Score
1'	191.019	Citric acid	173.0087, 129.0193, 87.0087, 73.0295, 59.0139	0.30	94.67
2'	175.061	2-Isopropylmalic acid	135.0463, 134.0385, 115.0407, 113.0595, 85.0663	0.09	95.63
3'	373.151	7-deoxyloganin	165.0926, 113.0257, 85.0274	-0.95	99.21
4'	431.192	Vomifoliol hexoside (formate adduct)	385, 223, 205, 153	1.01	98.95
5'	373.151	Wine lactone hexoside	327.1483, 166.0955, 165.0924, 113.0250, 101.0247, 89.0251, 85.0292, 73.0297, 71.0148, 59.0148	0.59	97.62
6'	187.098	Azelaic acid	125.0973, 123.0809, 97.0659, 95.0523, 80.0273, 57.0343	-2.01	98.66

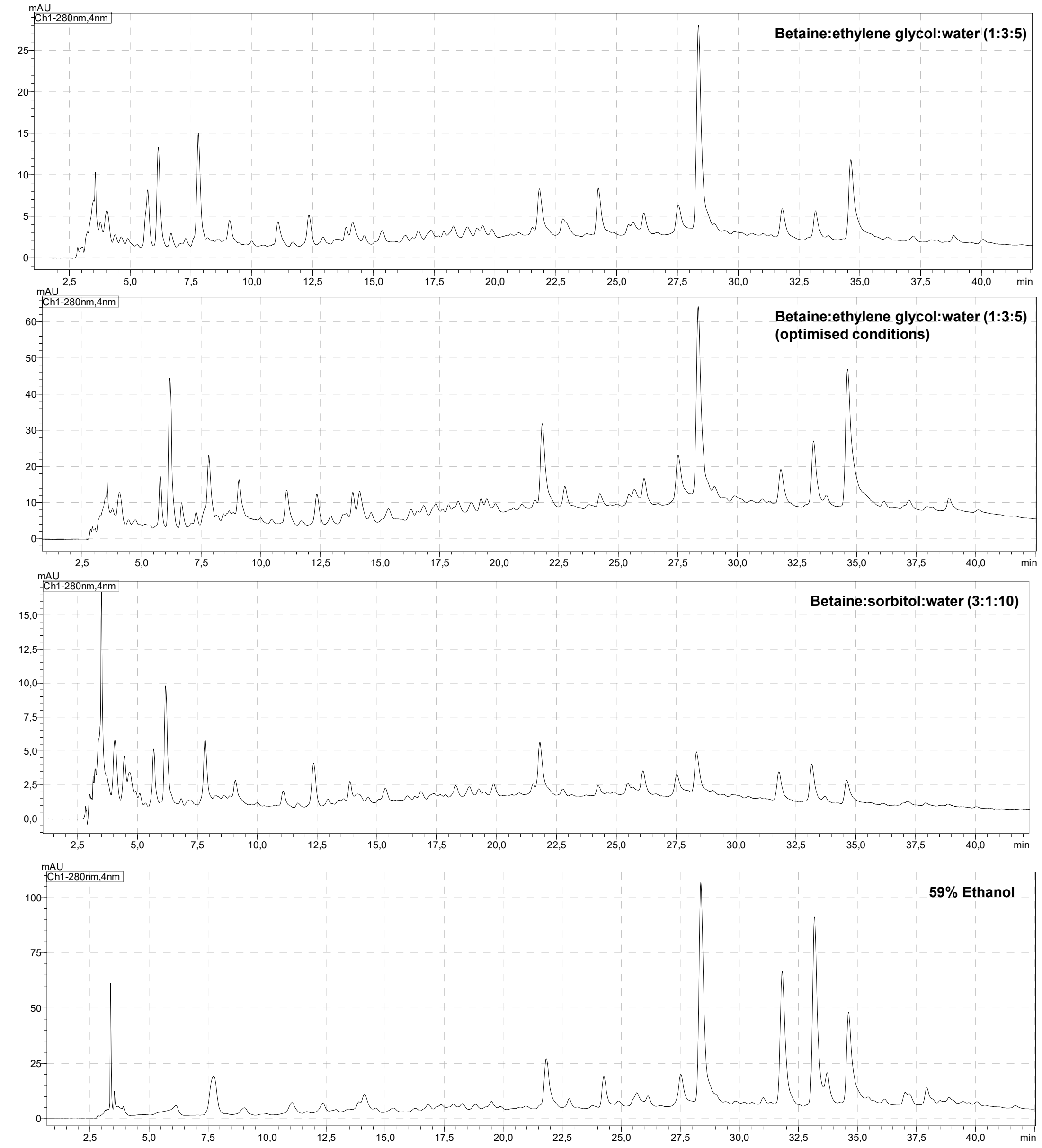

Figure S1. Chromatograms (280 nm) of the DES 3 (betaine:ethylene glycol:water, 1:3:5) extracts, before and after optimisation, DES 9 (betaine:sorbitol:water, 3:1:10) extract and the 59% ethanolic extract.

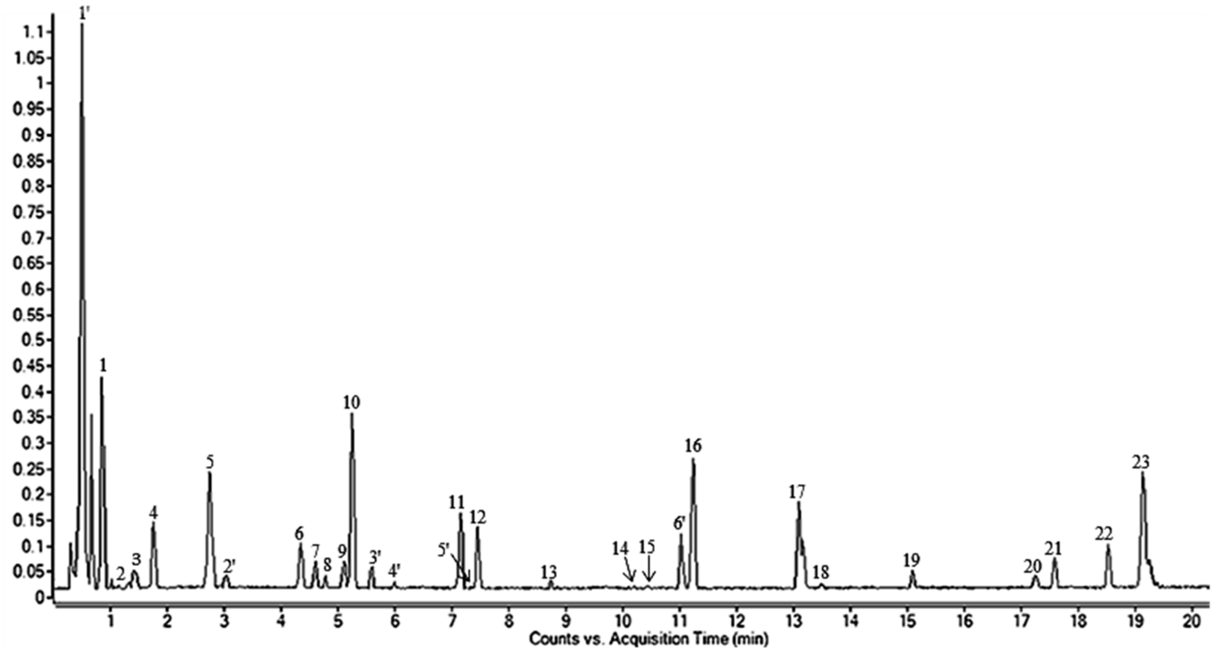

Figure S2. Base peak chromatogram of the DES 3 (betaine:ethylene glycol:water, 1:3:5) extract obtained at optimal conditions (80 °C, 127 min, and a L/S ratio of 10:1). Table 4 shows the numbering for each compound.
